# Chemotherapy and Dietary Phytochemical Agents

**DOI:** 10.1155/2012/282570

**Published:** 2012-12-20

**Authors:** Katrin Sak

**Affiliations:** NGO Praeventio, Näituse 22-3, 50407 Tartu, Estonia

## Abstract

Chemotherapy has been used for cancer treatment already for almost 70 years by targeting the proliferation potential and metastasising ability of tumour cells. Despite the progress made in the development of potent chemotherapy drugs, their toxicity to normal tissues and adverse side effects in multiple organ systems as well as drug resistance have remained the major obstacles for the successful clinical use. Cytotoxic agents decrease considerably the quality of life of cancer patients manifesting as acute complaints and impacting the life of survivors also for years after the treatment. Toxicity often limits the usefulness of anticancer agents being also the reason why many patients discontinue the treatment. The nutritional approach may be the means of helping to raise cancer therapy to a new level of success as supplementing or supporting the body with natural phytochemicals cannot only reduce adverse side effects but improve also the effectiveness of chemotherapeutics. Various plant-derived compounds improve the efficiency of cytotoxic agents, decrease their resistance, lower and alleviate toxic side effects, reduce the risk of tumour lysis syndrome, and detoxify the body of chemotherapeutics. The personalised approach using various phytochemicals provides thus a new dimension to the standard cancer therapy for improving its outcome in a complex and complementary way.

## 1. Introduction 

Chemotherapy is one of the principal modes of the treatment of cancer patients [1]. It was first used to treat advanced lymphoma in the late 1940s after it became known that the use of mustard gas in the World War I caused leukopenia [2, 3]. Shortly after the World War II, it was also found that folic acid stimulates the proliferation of acute lymphoblastic leukaemia cells and antagonistic analogues to folic acid, first aminopterin and then amethopterin (now known as methotrexate) induced the remission in children with acute lymphoblastic leukaemia [4]. Although the role of chemotherapy in the treatment of common, epithelial malignancies was limited to the treatment of symptomatic metastatic disease for almost 30 years, nowadays cancer chemotherapy has three main applications: it is curative for a small number of malignancies including childhood leukaemia, Hodgkin's and non-Hodgkin's lymphoma, and germ cell malignancies; it has a palliative role for most metastatic epithelial malignancies; and it has an adjuvant role in several types of resected epithelial malignancies [3]. 

 By definition, chemotherapy treatment should interfere with the biochemical program that is involved or committed to cellular replication and cause selective cell death. At that, the host cell should be able to adapt and recover from toxicity [2]. Many chemotherapeutic agents kill cancer cells oxidatively via the production of reactive oxygen species and the induction of either apoptosis or necrosis of tumorous cells [2, 5, 6]; whereas others act on various components of cellular metabolism influencing activities of different enzymes needful for cell division.

 Cancer treatment is targeted at its proliferation potential and its ability to metastasise; hence, the majority of chemotherapy drugs take advantage of the fact that cancer cells divide rapidly [7]. Chemotherapy agents can be divided into several categories based on the factors such as how they work, their chemical structure, and their relationship to another drug. The most important categories of chemotherapeutics include alkylating agents (e.g., cyclophosphamide, ifosfamide, melphalan, busulfan), antimetabolites (e.g., 5-fluorouracil, capecitabine, methotrexate, gemcitabine), antitumour antibiotics (e.g., daunorubicin, doxorubicin, epirubicin), topoisomerase inhibitors (e.g., topotecan, irinotecan, etoposide, teniposide), and mitotic inhibitors (e.g., paclitaxel, docetaxel, vinblastine, vincristine) [8, 9]. Most chemotherapeutic drugs target the cell cycle machinery relying on the difference in the frequency of cell division to differentiate between the cancer clones and normal cells. Within this process slow-growing cancer clones will survive and evolve into new fast growing strains. Chemotherapy is able to kill off most of the susceptible tumorous cells succeeding to send cancer into remission for weeks or months after which it reemerges as a more aggressive organism [10, 11]. In fact, the more chemotherapy is given, the higher is the aggressiveness of relapse. In these cases, chemotherapy may indirectly select the most resistant mutant cell for clonal expansion [2]. 

 Cancer is a highly heterogeneous disease, especially in its advanced forms [2], and such heterogeneity gives it an advantage to survive under selection pressure of drugs [12]. Moreover, the unique characteristics of tumour microenvironment (hypoxia, low extracellular pH, high interstitial fluid pressure), developed at least in part as a result of the malformed tumour vasculature, act as barriers to chemotherapy impairing the transport and delivery of circulating therapeutic molecules in tumour tissue [13–16]. Cancer hypoxia can thus reduce the effectiveness of drugs [13, 15, 17]. Another major obstacle of conventional chemotherapy proceeds from the drug resistance [1, 12, 18, 19]. Some tumours are intrinsically resistant to certain drugs, whereas others can acquire resistance after treatment [1, 18–20]. Cancer cells can often develop resistance not only to the agent, which they have been exposed to, but also to other drugs and chemicals that they have not encountered. There are a number of mechanisms mediating such multidrug resistance [2, 20, 21]. Upregulation of drug efflux ATP-binding cassette (ABC) transporters, such as P-glycoprotein (P-gp), multidrug resistance protein 1 (MRP1), and breast cancer resistance protein (BCRP), may be responsible for the resistance to many chemotherapeutics affecting disposition of these drugs in the tumour cells and modifying seriously the clinical outcome [1, 20, 22, 23]. Although the first human ABC drug transporter P-gp was identified already for more than 35 years ago, there are still no clinically applicable inhibitors of ABC transporters available to date and this failure is largely due to the unfavourable toxic side effects of tested chemical compounds. Novel chemosensitisers as inhibitors of different MDR-linked ABC transporters are actively explored turning increasing attention also to various natural products [23, 24]. 

## 2. Factors Affecting the Effectiveness of Chemotherapy

Effectiveness of chemotherapy depends on various factors, including properties of cancer cells (if tumour is hypoxic or mitochondrial function is severely compromised, or the number of mitochondria within the cancer cell is low, chemotherapy will be of limited value, only increasing the clonal selection of the most resistant and possibly also the most aggressive cancer phenotype [2]); tumour size (the microscopic form of tumour is much more successfully treated than macroscopic cancer [2]); number of chemotherapy cycles [25]; administrating polychemotherapy *versus* monotherapy (polychemotherapy may be more active than single agent, whereas the order of administration of drugs as well as their time schedule is also important: combining drugs with different modes of action may lead to enhanced or even synergistic antitumour effects without injuring the host [25–28]); multitargeted approach (targeting both the cancer cell and its microenvironment might increase the treatment efficiency [29]); and the phase of the circadian cycle [30]. Circadian periodicity in cell proliferation provides an opportunity to improve the tolerability or efficiency (or both) of anticancer treatment by targeting its timing at certain stages of the circadian system of the host or the tumour [30–37]. It has been shown in animal studies that the toxicity and efficacy of more than 30 anticancer agents vary by more than 50% depending on the circadian time when the agent is given [2, 31, 32, 35, 36]. The peak time of drug delivery to achieve optimal tolerability is characteristic for each chemotherapeutic agent and the circadian time of administration of cancer chemotherapeutic agents when they are best tolerated also usually shows the highest efficacy against the tumour [31, 35, 37]. Such chronotherapy may have a significant impact on the treatment success and can ultimately lead to a better tumour management [33, 35, 37]. The patient's gender could also have an essential role in the determination of optimal chronotherapeutic schedule [2, 36]. 

Cancer is a systemic and not a local disease [2]. Metastatic spread of tumour cells may take place already at an early stage of the malignancy; however, little is known about the tumour-biological parameters of such disseminated cells [38]. Metastatic foci might vary with genotype, phenotype, and drug response [21], whereas expression profiles and signalling pathways between primary tumour and metastatic tissue can be different [27, 38]. The considerable number of adjuvant therapy failures may be explained by such molecular differences between the surgically removed and histopathologically examined primary tumour cells and the remaining disseminated tumour cells [38]. Namely, the latest would be the real targets for any systemic therapy to prevent them forming a clinically relevant metastatic disease [21, 38].

## 3. Typical Side Effects of Chemotherapy

Although the desired goal of chemotherapy is to eliminate the tumour cells, diverse ranges of normal cell types are also affected, leading to many adverse side effects in multiple organ systems [5, 39–43]. Such debilitating effects are a major clinical problem [44], whereas the toxicity often limits the usefulness of anticancer agents [44, 45]. 

 Knowing how the chemotherapy agent works is important in predicting its side effects. For instance, treatment with alkylating agents and topoisomerase II inhibitors increases the risk of secondary cancer (acute leukaemia); anthracyclines (like doxorubicin) induce cardiotoxicity; and mitotic inhibitors have the potential to cause peripheral nerve damage [6]. 

The most common acute complaints of cancer patients undergoing cytotoxic therapy are fatigue, nausea, vomiting, malaise, diarrhoea, mucositis, pain, rashes, infections, headaches, and other problems [2, 42, 44, 46, 47]. Normal haematopoietic cells, intestinal epithelial cells, and hair matrix keratinocytes are often susceptible to the toxic effects of anticancer agents [44]. Over 75% of cancer patients suffer from therapy-associated fatigue; however, only about one-third of treating physicians recognise this problem [42]. Fatigue is related to a reduced mitochondrial function of tumorous cells and is often a significant reason why patients discontinue the treatment [42, 48]. Thus, cytotoxic agents decrease considerably the quality of life of patients [43, 48, 49]. Through the induction of nausea and vomiting, difficulties in swallowing, dry mouth, alterations in taste and smell, depression, poor energy, and aversion to food cytotoxic drugs affect also the nutritional status of patients [2, 47] showing that chemotherapy is a sufficient stressor in causing malnutrition. It must be appreciated that malnutrition is the reason why majority of the cancer patients die [2, 50]. 

Most cytotoxic drugs have immune suppressive side effects. Many chemotherapeutics kill dividing haematopoietic cells manifesting as profound neutropenia and cytopenia resulting in decreased immunity, increased susceptibility to infections, and elevated risk of bleeding [2, 3]. Coming from the requirement of the bone marrow to repopulate white cells and platelets in the blood, drugs are often administered episodically followed by the drug-free intervals of 2-3 weeks. Such scheme helps to minimise the chance of infection or bleeding but allows also the tumour to recover [51, 52]. 

Cancer patients frequently complain of neurological side effects [53]. Such effects range from abnormalities in brain volume and integrity detected by magnetic resonance imaging on patients after chemotherapy to different clinical symptoms; manifesting acutely or as delayed neurotoxicities only becoming apparent years after treatment [40, 41, 54]. Neurological complications include memory loss and cognitive dysfunction, seizures, vision loss, dementia, leukoencephalopathy, cerebral infarctions, and other problems [41, 53, 54]. These symptoms are commonly referred to as Chemo Brain [53, 55] affecting some 4–75% of cancer patients following with chemotherapy [55]. Nearly all frequently used chemotherapeutic agents can cause adverse neurological effects [40, 41]. Chemotherapy-induced cognitive changes might be associated with neurotoxic effects of inflammation and cytokine deregulation. Treatment-induced changes in the level of oestrogen and testosterone can be related to cognitive decline. Also, corticosteroids, which are commonly used as part of chemotherapy regimens or to manage side effects such as nausea, alter neuroendocrine functioning and cognition [40]. 

The most common long-term health problems of adjuvant chemotherapy include poor memory and concentration, visual deterioration, musculoskeletal complaints including early onset osteoporosis, poor sleep patterns, skin changes, sexual dysfunction, and chronic fatigue [3]. This complex of problems is suggestive of accelerated aging leading potentially to early onset frailty [3, 40]. Such long-term toxicity can have an impact on the quality of life of cancer survivals that could last for years [3]. 

Most chemotherapy drugs are genotoxic likely causing epigenetic and genetic damage [2, 44, 53]. Many cancer patients encounter thus the problem of developing second malignancies as a result of treatment [44, 48, 53]. The common secondary tumours are a variety of acute leukaemias and non-Hodgkin's lymphomas, less common are carcinomas of the urinary bladder and other malignancies, which are usually refractory to treatment. Secondary leukaemias constitute approximately 10% of all leukaemias having the average latent interval from the diagnosis and treatment of the primary neoplasm to development of acute leukaemia for four to six years. The International Agency for Research on Cancer (IARC) has identified 20 single chemotherapeutic agents or regimens which cause cancer in humans and about 50 others that are suspect [2]. Chemotherapy-associated immunosuppression can result in an increased rate of infection by oncogenic viruses which further increase the risk of secondary cancers [2]. 

Current treatment protocols often apply multiagent chemotherapy [54] and this may even increase the extent of adverse side effects [50]. Several serious complications can cause discontinuation of therapy, prolong the duration of stay in hospitals, and may affect the overall prognosis and outcome of the disease [50]. It is important to bear in mind that, in general, older cancer patients are more susceptible to treatment-related complications than younger individuals [56]. One of the most critical and life-threatening adverse conditions of chemotherapy needing immediate intervention is tumour lysis syndrome. It results from the massive and abrupt destruction and lysis of malignant cells and subsequent release of intracellular ions and metabolites into the bloodstream leading to hyperuricemia, hyperphosphatemia, hypocalcemia, and hyperkalemia [57, 58]. This syndrome is accompanied by renal failure and metabolic acidosis increasing the risk of death [57]. Although the tumour lysis syndrome is most frequently observed in patients with haematologic malignancies, it may also occur in solid tumours [57, 58]. These malignancies share the characteristics of a high proliferative rate, large tumour burden, or high sensitivity to cytotoxic therapy and in principle, any tumour that is highly responsive to chemotherapeutic drugs, particularly if the cancer cells die through the necrotic pathway, can give rise to this severe metabolic syndrome [2, 58]. Most of the complications can be readily managed when they are recognised early; however, delay in recognition and treatment of metabolic abnormalities may become fatal [58]. 

## 4. Nutritional Approach in Chemotherapy

The efficacy of standard oncologic therapies (involving a combination of surgery, multiple chemotherapeutic agents, and ionising radiation) has reached a plateau for most of solid tumours [48]. General protocol with these therapies is just to follow a watch-and-wait strategy after the therapeutic administration is concluded [9]. However, this is a period when supplemental therapies are highly indicated to result in a higher percentage of successful outcomes [9]. Cancer patient must be stocked up with needful nutrients already before receiving treatment in order to ensure the best health possible before undergoing chemotherapy [59]. The adverse side effects of anticancer agents exacerbate the nutritional problems and the proper nutrition should be an integral part of the treatment program of every cancer patients [47]. Dietary components may act as adaptogens protecting organism from the adverse effects of intervention or as modifiers of biologic response [60] exerting additive or even synergistic effects with pharmaceutical agents [61].

 Thus, nutritional approach may be the means of helping to raise cancer therapy to a new level of success, whereas supplementing or supporting the body with natural compounds may be the answer in not only reducing the side effects but also improving the effectiveness of chemotherapy [2]. At the same time, it is important to bear in mind that some nutrients can affect the treatment outcome also adversely by attenuating or inhibiting the therapeutic effect of certain drugs. Optimal dietary regimen of cancer patients should thus be prescribed by a nutritional oncologist considering each individual case separately, whereas the consumption of supplements on patient's own initiative may be dangerous and should be avoided. 

## 5. Purposes of Nutritional Intervention

Intervention with dietary agents in chemotherapy has several aims. It increases the efficacy of treatment and decreases its side effects, improves cancer killing through apoptosis rather than necrosis, reduces drug resistance or increases drug accumulation within cancer cells, detoxifies body of chemotherapeutics, decreases weight loss and malnutrition, improves the quality of life, and reduces severity of comorbid conditions. 

Dietary compounds may enhance the efficacy of cancer therapeutics by modifying the activity of key cell proliferation and survival pathways [48, 61, 62]. Multiple antioxidants are effective in increasing the tumour response to chemotherapy improving also the survival time of patients [9, 48, 59]. Nutrients can be used also to mitigate the toxicity of chemotherapy [46, 48, 62]. Reduction in adverse effects of anticancer agents has been shown when given concurrently with antioxidants [9, 48, 59]. Lowering the side effects may permit to safely administer a higher and possibly more effective dose of chemotherapeutic drugs [2]. Combination of nutrients has generally a better effect getting the optimum therapeutic response and reducing adverse side effects than different nutrients separately. However, possible interactions between the dietary components during chemotherapy need to be carefully controlled as some supplements can abolish the beneficial effects of others [2]. 

Oxidant stress shifts the mechanisms of cell killing away from apoptosis to necrosis inducing inflammation and promoting cancer progression. Necrosis will increase also the risk of developing tumour lysis syndrome. Regulating the oxidant state with specific antioxidants, supporting mitochondrial function, and increasing their numbers within cancer cells can promote the apoptosis-induced death of chemotherapy. This reduces the side effects associated with chemotherapy and may also lower the resistance to anticancer agents and the progression of tumour [2, 9].

Drug resistance is one of the primary obstacles of successful chemotherapy. Inhibiting the function of ABC transporters, such as P-gp and MRP-s, may lead to overcoming drug resistance providing a possible mechanism to improve chemotherapeutic effects [2, 20]. Although co-administration of transport inhibitors together with the actual anticancer drug may enhance drug penetration into the tumour [24], no real solution to multidrug resistance has been found to date and no chemical P-gp inhibitors with clinically satisfying results have been described mainly due to their high toxicity [23, 24]. Therefore, several novel compounds including those isolated from natural sources (or the so-called “fourth generation chemosensitisers”) are actively explored and may hopefully provide some solution to overcome these problems [20, 23, 24]. 

The body needs to be detoxified of chemotherapeutics after the treatment is completed. Many of the anticancer agents are carcinogenic themselves and the patients may suffer secondary cancers following primary remission from the initial tumour [2]. 

Nutritional status of cancer patients is associated with the outcome of malignant disease while weight loss is related to shortened survival and lowered response to chemotherapy [2, 47]. Malnutrition is the cause of death for the majority of cancer patients [2]. Therefore, nutritional support enhances the chances of complete remission of disease and improves the quality of life [47]. 

Cancer patients may be antioxidant deficient and chemotherapy further generates free radicals that cause oxidative damage in different organs. It is important to keep comorbid conditions in mind considering the state of the entire patient and not just to focus on the cancer. The antioxidant status of the patient can remain depressed for some months after the cancer treatment and nutritional support helps to ensure the best possible general health state [59]. 

## 6. Plant-Derived Dietary Agents in Chemotherapy

The optimal nutritional program proceeds from each individual case and considers the needs of a certain patient. Dietary phytochemical agents influence various aspects of chemotherapy treatment and their involvement in the cure of cancer patients is absolutely indicated and needful. Different natural compounds can improve efficiency of chemotherapeutic agents, decrease the resistance of chemotherapeutic drugs, lower and alleviate the adverse side effects of chemotherapy, reduce the risk of tumour lysis syndrome, and detoxify the body of chemotherapeutics. At the same time, it is important to be aware that some phytochemical agents can have also toxic effects and influence the treatment results adversely. 

Effects of plant-derived dietary agents on treatment output have been intensively explored; however, most of the studies about interactions between dietary phytochemical agents and chemotherapy drugs are done using either *in vitro* cell systems or *in vivo* animal experiments (see Table 1). Therefore and before more clinical data will be available, some precaution must be taken in transferring these results directly to the patients.

Various plant-derived agents like genistein, curcumin, epigallocatechin gallate (EGCG), resveratrol, indole-3-carbinol, and proanthocyanidin have been shown to be able to affect the efficacy of traditional chemotherapeutic agents [48, 61]. Supplementation with bromelain can also increase the cytotoxic activity of several drugs and reduce inflammatory responses [2, 42]. 

Saponins from Chinese ginseng enhance the therapeutic effect and behave as adaptogens reducing haematopoietic complications induced by systemic chemotherapy [60, 78]. Laboratory experiments have shown that saponins are able to protect against cyclophosphamide- (CY-) induced genotoxicity and apoptosis in bone marrow cells and peripheral lymphocytes [78], some ginsenosides are able to sensitise drug-resistant breast cancer cells to paclitaxel [63], and American ginseng can attenuate nausea and vomiting induced by cisplatin while enhancing its antiproliferative effect on human breast cancer cells [63]. Notoginseng extract can synergistically increase the antiproliferative effect of 5-fluorouracil (5-FU) in human colorectal cancer cell line making it possible to reduce the dose of 5-FU in combination with notoginseng and thereby further decrease its dose-related toxicity [63]. 

Polyphenol quercetin is able to potentiate the cytotoxic effect of cisplatin while protecting normal renal cells from cisplatin toxicity. Quercetin can work synergistically with busulfan against human leukaemia cells and with doxorubicin in cultured multidrug-resistant human breast cancer cells. It increases cytotoxic effect of CY and decreases resistance to gemcitabine, topotecan, vincristine, tamoxifen, paclitaxel, and doxorubicin [9, 20, 68, 71, 90]. Quercetin stimulates also the activity of macrophages thereby further improving the chemotherapeutical efficiency [2]. 

Catechins from green tea can increase the therapeutic effect of doxorubicin in drug-resistant tumours in animal studies [68]. Administration of green tea increases the concentration of this chemotherapeutic agent in tumour but not in normal tissue enhancing its antitumour activity [9]. 

 Curcumin can also enhance the tumoricidal efficacy of cytotoxic chemotherapy [66] and behave as adaptogen [60]. 

However, administration of soy isoflavones has led to contradictory results emphasising the necessity to maintain caution in excessive consumption of dietary agents and supplements during treatment with chemotherapy. While genistein can potentiate the action of tamoxifen to inhibit the tumour cell growth in oestrogen receptor-negative human breast cancer cells, this isoflavone can attenuate the tumoricidal activity of tamoxifen in oestrogen-dependent breast carcinoma cells (see Table 1). Also, as a combination of daidzein with tamoxifen produces increased protection against mammary carcinogenesis [9, 75], supplementation of genistein can negate the inhibitory effect of tamoxifen on tumour burden [75]. The therapeutic effect of tamoxifen can be fully blocked also by flavonoid tangeretin [9, 68] and until the interactions between flavonoids and tamoxifen will be more clear, therapeutic doses of flavonoid compounds should be avoided in nutritional supporting programs of breast cancer patients treated with tamoxifen [9].

Several naturally occurring plant agents like flavonoids can enhance the drug bioavailability by inhibiting ATP transporters-mediated drug efflux *in vitro*, suggesting that such interactions could occur also *in vivo* [20]. It is recommended therefore that diet of cancer patients treated with chemotherapy should be rich in herbal constituents (including quercetin, kaempferol, naringenin, silymarin, catechins), fruits and berries (e.g., grapefruit, orange, apricot, strawberry), and spices (mint, rosemary, curcumin, garlic, ginseng, piper nigrum, onion) [20]. 

To ensure the removal of toxic waste products, it is important that phase II detoxification enzymes are stimulated and restored. For this purpose, cancer patients should increase the intake of isothiocyanates (found in various cruciferous vegetables, particularly Brussels sprouts and red cabbage), naringin (in grapefruit), and parsley and spice their food with curcumin [2]. Detoxification program should be undertaken to eliminate the potentially mutagenic chemotherapeutic agents from organism. Various dietary compounds contribute to induce specific detoxification pathways whereas such multifunctional inducers include many of the flavonoid molecules found in various fruits and vegetables [22]. Increase in activity of phase II enzymes supports the better detoxification and it can be achieved by substances abundantly found in red grapes, garlic oil, rosemary, soy, cabbage, Brussels sprouts, and broccoli [2, 22]. 

## 7. Role of Vitamins in Chemotherapy

It is becoming more and more clear that vitamins can reduce the side effects of chemotherapy treatment. Thus, vitamin A reduces adverse effects of cyclophosphamide in rats [48] and its coadministration with methotrexate ameliorates intestinal damage in studies of mice without any inhibition of antitumour activity [9, 68]. Also folic acid (or vitamin B9) supplementation during chemotherapy may lead to reduction of toxicity [2]; however, it should not be added to methotrexate treatment, as this anticancer drug is a folic acid inhibitor [9]. Oncologists should therefore advise their patients who undergo chemotherapy with antifolate drugs (such as methotrexate) not to take folate supplements or consume folate-rich food in excess. A folate status of the patient may influence the response to methotrexate and can possibly support cancer growth [91]. 

 Vitamin C reduces the adverse effects of some chemotherapeutic agents on normal cells, such as those from doxorubicin. This vitamin can lower tamoxifen-induced hyperlipidemia in women with breast cancer and has been shown to reduce bleomycin-induced chromosomal breakage in human lymphoid cells *in vitro* [48]. In experiments carried out with mice and guinea pigs, vitamin C led to a reduction in cardiotoxicity of doxorubicin without any reduce in antitumour efficacy [9]. 

 Animal studies carried out mainly with rats and rabbits have shown the ability of vitamin E to reduce bleomycin-induced lung fibrosis, doxorubicin-induced cardiac toxicity, and doxorubicin-induced skin necrosis. It lowers also doxorubicin-induced toxicity in liver, kidney, and intestinal mucosa. Coadministration of vitamin E and vitamin C can reduce the tamoxifen-induced hyperlipidemia in women with breast cancer [48]. The most effective form of vitamin E is *α*-tocopheryl succinate that can be used as an adjunct to standard cancer therapies in order to improve their efficacy on tumour cells while protecting normal cells against some of their toxicities [92, 93]. 

 Vitamin K3 can act synergistically when combined with several conventional chemotherapeutic agents [94]. However, it is important to keep in mind that while this vitamin is able to induce cell cycle arrest at the G2/M phase, it is useful as an enhancer of G2 phase-dependent chemotherapeutic drug etoposide, whereas reduces the cytotoxic activity of S-phase-dependent agent irinotecan [95]. 

 Last but not least, vitamin-like compound coenzyme Q10 has also been shown to ameliorate the side effects of chemotherapies [96]. It helped to prevent doxorubicin-associated cardiotoxicity in a small human study whereas the diarrhoea and stomatitis were also significantly reduced. This compound can lower also daunorubicin-induced adverse events in leukaemia patients without any reduce in the therapeutic benefit [9, 68]. 

 These data demonstrate the potential of dietary agents to reduce the symptoms of adverse side effects of chemotherapy treatment and relieve the situation of patients, improving their quality of life. Therefore, nutritional support programme built upon the personal necessities and clinical purposes is an absolutely important part of treatment scheme for all cancer patients. 

## 8. Potential Problems Prescribing Phytochemical Agents to Chemotherapy Patients 

Despite a huge amount of studies published about the benefits of nutrient supplementation in chemotherapy there are still a lot of sceptics and opponents among both physicians as well as patients. On the one hand, this can be due to the lack of knowledge; on the other hand, spread of misconceptions and false beliefs also play an important role. 

It is estimate that roughly 50% of cancer patients use some kind of dietary supplements [50, 97]. Many standard chemotherapeutic agents mediate their cytotoxic effects by generating excessive amounts of free radicals [5, 6, 48, 97] and a long-standing concern with the use of phytochemical antioxidants has been comprised of their potential theoretical intervention to the effectiveness of chemotherapy [5, 6, 9, 68]. It might be expected that antioxidants, quenching the reactive oxygen species, can protect tumour cells as well as healthy cells from oxidative damage generated by chemotherapeutic drugs [5, 97–99]. This could lead to reduction in effectiveness of cytotoxic therapy and many oncologists are of the opinion that taking antioxidants concurrently with chemotherapeutic drugs might be harmful [5, 48, 59, 97, 99, 100]. However, studies involving thousands of patients have demonstrated that antioxidants and other herbal nutrients do not interfere with chemotherapy, but instead provide a wide range of beneficial effects [5, 101]. Antioxidants can protect normal tissues from chemotherapy-induced damage without decreasing oncological efficacy [5, 9, 98]. These nutrients reduce the treatment-related side effects and lower the risk and severity of comorbidities [5, 6, 9, 59, 98, 101, 102] and improve the quality of life and overall survival of cancer patients [9, 59, 101]. Some of antioxidants may enhance the effects of cytotoxic regimens improving the response rate of tumour to chemotherapeutic agents [9, 59, 98, 101, 102]. 

Currently, a number of scientists hold the position that antioxidants do not interfere with chemotherapy and at commonly used dosages they enhance the success of the treatment. Except for some specific interactions (including flavonoids with tamoxifen) [5, 9], the weight of the literature supports the use of antioxidants during the treatment and also in maintenance phase [59]. It is better to consume combinations of food-based antioxidants rather than synthetic single compounds (vitamins) [59], whereas the form of a particular antioxidant as well as dosages and dose schedules are also important [103]. Physicians need to remain aware of the large body of evidence showing beneficial effects of antioxidants on the outcomes of chemotherapy treatment [9] and apply this knowledge in their everyday clinical practice. 

## 9. Conclusions and Further Perspectives

Developing the rational strategies for fighting the cancer requires first a clear understanding of the causes and pathogenesis of the disease [104]. This comprises a multidimensional approach of the studies at genomic, proteomic, and metabolomic level considering both the individual variances as well as interindividual differences. Harnessing the mutations that cancer cells need to promote their pathological survival and expansion will be the basis of further therapeutic strategies [19, 44]. In the future, each patient should have his own unique chemotherapy protocol, which improves the therapeutic quality by selecting and prescribing well-matched drugs and avoiding ineffective ones [21]. Applying such individualised chemotherapeutics through a personalised chronotherapy regime will further improve the final outcome [2, 36]. This will be accompanied by the identification and testing of novel more specific and selective drugs either via synthetic routes or by purifying from herbal sources [39]. 

 Although the novel chemotherapeutic agents will be more and more effective against the tumour cells, their toxicity to normal tissues as well as drug resistance remains the major obstacles for clinical use. Personalised approach using various phytochemical compounds provides a new dimension to the standard cancer therapy for improving its outcome in a complex and complementary way.

## Figures and Tables

**Table 1:** Examples of effects of dietary phytochemical agents on chemotherapy.

Compound	Dietary source	Chemotherapy drug	Effect	Biological system	Reference
Influence on treatment efficacy					

Ginsenosides		Cisplatin	Enhancement of drug-induced antiproliferative effect	Human breast carcinoma MCF-7 cells	[63, 64]
	Ginseng	5-Fluorouracil	Increase in antiproliferative effect	Human colorectal cancer HCT-116 cells	[63, 65]
Curcumin	Turmeric	Vinorelbine	Enhancement of chemotherapeutic efficacy	Human squamous cell lung carcinoma H520 cells	[66, 67]
Catechins/theanine	Green tea	Doxorubicin	Enhancement of antitumour activity	Ehrlich ascites carcinoma and M5076 ovarian sarcoma tumour-bearing mice	[9, 68–70]
Quercetin			Increase in reduction of tumour growth	Mice bearing human tumour xenografts	[71]
		Cisplatin	Potentiation of cytotoxic effect	Human ovarian and endometrial cancer cell lines	[71]
	Many foods such as onions, apples, berries, and tea	Doxorubicin	Potentiation of growth-inhibitory activity	Doxorubicin-resistant human breast tumour MCF-7 cells	[68, 71, 72]
		Busulfan	Synergistic antiproliferative activity	Human leukaemia K562 cells	[71, 73]
Genistein		Cisplatin	Increased cytotoxic effect	Cisplatin-sensitive and cisplatin-resistant human 2008 ovarian carcinoma cells	[9, 71]
			Attenuation of inhibitory effect of tamoxifen on tumour cell growth	Oestrogen-dependent human breast cancer MCF-7 cells	[9, 74]
	Soy foods	Tamoxifen	Attenuation of tamoxifen effect on reducing of tumour burden	Female Sprague-Dawley rats with induced mammary tumours	[75]
			Synergistic growth inhibition	Oestrogen receptor-negative human breast carcinoma MDA-MB-435 cells	[9, 76]
Daidzein	Soy foods	Tamoxifen	Improvement of drug activity to reduce tumour burden	Female Sprague-Dawley rats with induced mammary tumours	[9, 75]
Tangeretin	Tangerine and other citrus peels	Tamoxifen	Complete blocking of growth inhibitory effect of tamoxifen	Female nude mice inoculated with human MCF-7/6 mammary adenocarcinoma cells	[9, 68, 77]

Influence on side effects of chemotherapy					

Ginsenosides		Cyclophosphamide	Protection against drug-induced genotoxicity and apoptosis in bone marrow cells and peripheral lymphocytes	Mouse peripheral lymphocytes and bone marrow cells	[78, 79]
	Ginseng	Cisplatin	Attenuation of drug-induced nausea and vomiting	Rat model	[63, 80]
Quercetin	Many foods such as onions, apples, berries, and tea	Cisplatin	Protection of normal renal tubular cells from drug toxicity	Pig kidney tubular epithelial LLC-PK1 cells	[71, 81]

Influence on drug resistance					

Ginsenosides	Ginseng	Paclitaxel	Chemosensitisation	Multidrug-resistant breast cancer cells	[63, 82]
Catechins/theanine		Doxorubicin	Inhibition of drug efflux from tumour cells	Drug-resistant M5076 ovarian sarcoma tumour-bearing mice	[9, 68, 70]
	Green tea	Daunorubicin	Increase in drug accumulation in tumour cells	Multidrug-resistant P-gp overexpressing human epidermal carcinoma KB-C2 cells	[20, 83]
		Irinotecan, SN-38	Inhibiting drug transport into biliary elimination and prolonging half-lives in plasma	Male Sprague-Dawley rats	[20, 84]

Quercetin		Vincristine	Increase in drug uptake in tumour cells	Doxorubicin-resistant human myelogenous leukaemia K562 cells	[20, 85]
		Tamoxifen	Enhancement of drug bioavailability decreasing the efflux by MDR transporters	Female Sprague-Dawley rats	[20, 86]
		Paclitaxel	Enhancement of drug bioavailability	Male Sprague-Dawley rats	[20, 87]
	Many foods such as onions, apples, berries, and tea	Doxorubicin	Potentiation of antitumour effect reducing P-gp expression	Multidrug-resistant human breast cancer MCF-7 cells	[9, 20, 72]
		Topotecan	Chemosensitisation	Mouse fibrosarcoma WEHI-S cells	[71, 88]
		Gemcitabine	Chemosensitisation	Mouse fibrosarcoma WEHI-S cells	[71, 88]
Genistein	Soy foods	Paclitaxel	Enhancement in systemic exposure of drug	Male Sprague-Dawley rats	[20, 89]
